# Supplementary material for: A comparison of human and GPT-4 use of probabilistic phrases in a coordination game
Source: Sci Rep. 2024 Mar 21;14:6835. doi: 10.1038/s41598-024-56740-9 (PMC10958015; doi:10.1038/s41598-024-56740-9)
Supplement: Supplementary file 1 — Supplementary Information. [file 41598_2024_56740_MOESM1_ESM.docx]

Supplement to Maloney, Dal Martello, Fei & Ma, *A Comparison of Human and GPT-4 Use of Probabilistic Phrases in a Coordination Game.*

In each panel of Figures 2,4,5,6 we plotted a scatterplot of bivariate data. Analyses of this data were based on univariate linear model fits. We reported tests of the hypothesis that the Intercept of the fitted line is 0 and the hypothesis that the Slope is 1. Otherwise the fitted parameters give an indication of how the plotted data deviates from the identity line. These fitted parameters serve as summary statistics aiding in interpreting the data.

The hypothesis tests are t-tests and we report the estimated parameter (Intercept or Slope), the Student’s t-statistic, the degrees of freedom (DF) and the exact p-value.

When outliers are present in a panel of Figures 2,4,5 or 6 we report analyses with outliers excluded in the main text and with outliers excluded and included in this Supplement. The outliers in a figure panel (if there are any) are labeled by their probability phrases (Table 1) in red. All p-values less that 0.05 are marked with an asterisk.

Figure 2: Probability: Human vs GPT

Figure 2A: Human vs GPT: Probability Investment [outlier D excluded]

**Estimate t-value DF p-value**

**Intercept:** 1.5889 0.4205 22 0.3391

**Slope:** 0.8334 -2.5767 22 0.0086*

Figure 2A: Human vs GPT: Probability Investment [outlier D included]

**Estimate t-value DF p-value**

**Intercept:** 7.6934 1.3114 23 0.1013

**Slope:** 1.0283 0.2418 23 0.4055

Figure 2B: Hum vs GPT: Probability Medical [outlier N excluded]

**Estimate t-value DF p-value**

**Intercept:** 11.3487 3.4575 22 0.0011*

**Slope:** 0.8245 -2.9333 22 0.0038*

Figure 2B: Hum vs GPT: Probability Medical [outlier N included]

**Estimate t-value DF p-value**

**Intercept:** 14.7345 2.5335 23 0.0093*

**Slope:** 0.6937 -3.0117 23 0.0031*

Figure 4: Ambiguity: Human vs GPT

Figure 4A: Investment

**Estimate t-value DF p-value**

**Intercept:** 4.1688 0.7290 23 0.2367

**Slope:** 0.5302 -4.8357 23 0.0000*

Figure 4B: Medical

**Estimate t-value DF p-value**

**Intercept:** 5.8198 1.2861 23 0.1056

**Slope:** 0.7100 -3.4582 23 0.0011*

Figure 5: Probability across Context

Figure 5A: Human

**Estimate t-value DF p-value**

**Intercept:** -0.1467 -0.0407 23 0.4839

**Slope:** 0.9072 -1.4063 23 0.0865

Figure 5B: GPT [outliers D, N excluded]

**Estimate t-value DF p-value**

**Intercept:** -2.4102 -0.6084 21 0.2747

**Slope:** 0.9770 -0.3414 21 0.3681

Figure 5B: GPT [outliers D, N included]

**Estimate t-value DF p-value**

**Intercept:** 17.0476 2.4267 23 0.0117*

**Slope:** 0.7366 -2.1439 23 0.0214*

Figure 6: Ambiguity across Context

Figure 6A: Human

**Estimate t-value DF p-value**

**Intercept:** 0.0482 0.0206 23 0.4919

**Slope:** 0.8190 -3.4649 23 0.0010*

Figure 6B: GPT

**Estimate t-value DF p-value**

**Intercept:** 8.4247 2.2144 23 0.0185*

**Slope:** 0.9411 -0.8359 23 0.2059
